# Heterocellular gene signatures reveal luminal-A breast cancer heterogeneity and differential therapeutic responses

**DOI:** 10.1038/s41523-019-0116-8

**Published:** 2019-08-02

**Authors:** Pawan Poudel, Gift Nyamundanda, Yatish Patil, Maggie Chon U Cheang, Anguraj Sadanandam

**Affiliations:** 1 Division of Molecular Pathology, Institute of Cancer Research, London, UK; 2 Centre for Molecular Pathology, Royal Marsden Hospital, London, UK; 3 Division of Clinical Studies, Institute of Cancer Research, London, UK

**Keywords:** Tumour heterogeneity, Cancer genomics

## Abstract

Breast cancer is a highly heterogeneous disease. Although differences between intrinsic breast cancer subtypes have been well studied, heterogeneity within each subtype, especially luminal-A cancers, requires further interrogation to personalize disease management. Here, we applied well-characterized and cancer-associated heterocellular signatures representing stem, mesenchymal, stromal, immune, and epithelial cell types to breast cancer. This analysis stratified the luminal-A breast cancer samples into five subtypes with a majority of them enriched for a subtype (stem-like) that has increased stem and stromal cell gene signatures, representing potential luminal progenitor origin. The enrichment of immune checkpoint genes and other immune cell types in two (including stem-like) of the five heterocellular subtypes of luminal-A tumors suggest their potential response to immunotherapy. These immune-enriched subtypes of luminal-A tumors (containing only estrogen receptor positive samples) showed good or intermediate prognosis along with the two other differentiated subtypes as assessed using recurrence-free and distant metastasis-free patient survival outcomes. On the other hand, a partially differentiated subtype of luminal-A breast cancer with transit-amplifying colon-crypt characteristics showed poor prognosis. Furthermore, published luminal-A subtypes associated with specific somatic copy number alterations and mutations shared similar cellular and mutational characteristics to colorectal cancer subtypes where the heterocellular signatures were derived. These heterocellular subtypes reveal transcriptome and cell-type based heterogeneity of luminal-A and other breast cancer subtypes that may be useful for additional understanding of the cancer type and potential patient stratification and personalized medicine.

## Introduction

Breast cancer is the most common female malignancy worldwide. Breast cancers are clinically and molecularly heterogenous, with five to ten “intrinsic” subtypes now recognized based on gene expression or integrated molecular characteristics, respectively.^1,2^ Among the intrinsic gene expression breast cancer subtypes, luminal-A breast cancers represent the majority of estrogen receptor (ER) and progesterone receptor (PR) high/positive tumors.^1,3^ Although many luminal-A tumors are highly responsive to endocrine therapies like tamoxifen, a significant proportion possess intrinsic and/or acquired resistance.^4,5^ Even this relatively well-characterized breast cancer subtype possesses heterogeneity at the levels of hormone receptor expression,^6,7^ treatment response,^5^ and genetic variability^2,3^ that requires further understanding.

Ciriello et al.^3^ defined at least four genetic subtypes of luminal-A tumors involving mutations and somatic copy number alterations (CNAs) potentially associated with tamoxifen resistance. However, genetic changes alone do not explain the entire spectrum of luminal-A heterogeneity. The factors leading to tumor heterogeneity, including in luminal-A tumors, are complex and include interactions between different cell types and the tumor microenvironment along with the genetic changes present within the epithelial compartment.^8^ For instance, stroma containing cancer-associated fibroblasts (CAFs) is most associated with basal/claudin-low breast cancers.^9^ However, the exact role of stroma/CAFs in luminal-A breast cancers is unclear.

**Fig. 1:**
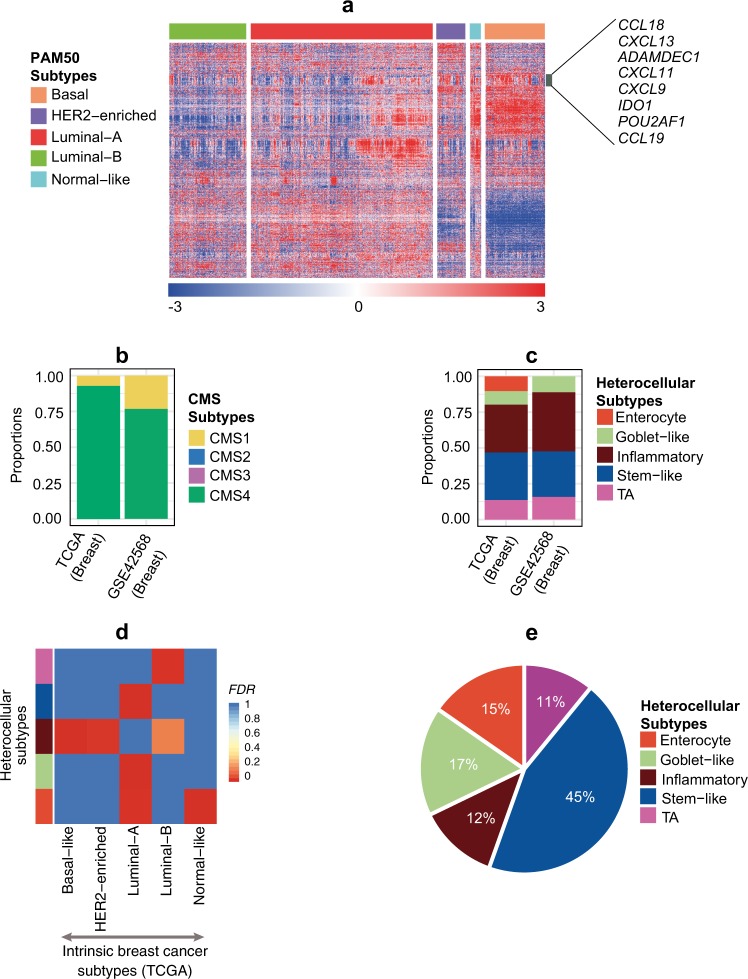
Association of breast cancer with heterocellular subtypes. **a** Heatmap showing the expression of the top highly variable genes (standard deviation; SD > 2), specifically immune genes, and their association with breast cancer subtype samples (*n* = 817) from TCGA.^23^ Highlighted genes represent selected immune specific genes that show high expression in multiple subtypes. **b** Proportion of CMS subtypes in multiple breast cancer data sets–TCGA^23^ (*n* = 671) and GSE42568^24^ (*n* = 69). **c** Proportion of heterocellular subtypes in multiple breast cancer data sets–TCGA^23^ (*n* = 407) and GSE42568^24^ (*n* = 63). Although heterocellular signatures were derived from entirely different cancer type (CRC), we observed that about half of the breast cancer samples were classified into all of the five heterocellular subtypes (stringent cutoff was used for mixed/low-confidence sample selection as discussed;^13^ Supplementary Table 1c). **d** Heatmap showing sample enrichment analysis using hypergeometric test-based FDR values comparing heterocellular subtypes (*y* axis) with intrinsic gene expression subtypes (*x* axis) in the TCGA^23^ breast cancer data set (*n* = 407; Supplementary Table 1e–g). **e** Pie chart showing proportions of different heterocellular subtypes in luminal-A breast cancer samples (total *n* = 202; enterocyte (*n* = 31), goblet-like (*n* = 34), inflammatory (*n* = 25), stem-like (*n* = 90), TA (*n* = 22); TCGA breast cancer^23^). Only those samples classified into subtypes with high confidence by the CMS and heterocellular classifiers were shown in **b**–**e**). Summary of low and high confidence samples for both subtype classifications are shown in Supplementary Tables 1a–d and 2a–d and described in Methods section

Moreover, the role of the immune microenvironment in luminal-A tumors requires further exploration. It is particularly important to understand luminal-A heterogeneity and drug resistance at the levels of the immune and stromal microenvironment. Unlike in colorectal and pancreatic cancers,^10–13^ no exclusive immune-enriched breast cancer subtype has been reported (to our knowledge). Nevertheless, immune-related genes are often expressed in different subtypes, including the luminal-A subtype (Fig. 1a), with signatures similar to those seen in one of the colorectal cancer (CRC) subtypes—consensus molecular subtypes (CMS)1/inflammatory.^11,13^ This prompted us to further interrogate molecular similarities between breast cancer and CRC.

We previously classified CRC into five CRCAssigner subtypes: inflammatory, enterocyte, goblet-like, stem-like, and transit-amplifying (TA).^11,14^ Later, we reconciled these five subtypes into four CMS1 to 4 using additional data from independently published CRC subtyping studies.^11,13,15–19^ CMS and CRCAssigner subtypes are >90% concordant with certain differences including that the enterocyte and TA subtypes were merged to form the CMS2 subtype.^13,20^ Most importantly, the immune-enriched groups (CMS1 and inflammatory) were similar. These CRCAssigner subtypes represent signatures related to stem, mesenchymal, and stromal cells forming the stem-like subtype, immune cells forming the inflammatory subtype, a partially differentiated state as the TA subtype, and a differentiated/secretory state as goblet-like and enterocyte subtypes.^11^ Therefore, we re-named the CRCAssigner subtypes as “heterocellular” subtypes in this study. Similar to the comparison of breast cancer subtypes to multiple cancer types,^21,22^ we sought to use our CRC heterocellular signatures as surrogates to re-characterize breast cancer subtypes, especially luminal-A breast cancers, and understand their phenotypes according to their differentiated, stem, fibroblast, and immune characteristics. This type of supervised analysis identifies low-frequent or rare intrinsic subtypes that are often difficult to characterize by unsupervised analysis. In addition, interesting sub-subtypes can be identified that we are reporting in this study for the luminal-A breast cancer subtype with potential personalized treatment associations.

## Results

### Association between breast cancer and heterocellular subtypes

To characterize the breast cancers using heterocellular subtypes, we applied the CMS classifier^13^ to two independent breast cancer data sets (The Cancer Genome Atlas; TCGA (*n* = 817)^23^ and GSE42568 (*n* = 104);^24^ Fig. 1b and Supplementary Tables 1a, b and 2a, b). Unexpectedly, the CMS classifier was only enriched for the CMS4 (mesenchymal; >75% of high confidence samples; see Methods section) subtype in these data sets, suggesting that this CMS classifier is specific to CRC and may not be applicable to breast cancer. Since our heterocellular (CRCAssigner) signature was derived earlier than and differently to the CMS and describes the phenotypic characteristics of normal colon-crypt cells including immune-enriched inflammatory cells,^11^ we applied this signature to the same data sets and observed that all five heterocellular subtypes were present in the TCGA breast cancer data set and four subtypes (except the CRC specialized subtype—enterocyte) in GSE42568 data set (Fig. 1c). There was a similar distribution of the four major subtypes (except enterocyte) in TCGA and GSE42568 data sets, with a variable proportion (between 0% and 10%) of the enterocyte subtype between these data sets. Correspondingly, enterocyte subtype was not present in normal breast tissue (Supplementary Figures 1a–c and Supplementary information). Here, only those samples with statistically high confidence of classification were considered (see Methods section; Fig. 1c; the dominant subtype distribution in mixed/low-confidence samples is shown Supplementary Figures 1d–f and Supplementary Tables 1c, d and 2c, d). This suggests that breast cancer has heterocellular features of different cell types (with variable proportions of enterocyte) that can be characterized with high confidence using our heterocellular signatures and subtypes.

We next sought to understand the relationship between the intrinsic breast cancer subtypes and heterocellular subtypes using hypergeometric sample enrichment analysis of the TCGA data set.^23^ The luminal-B intrinsic breast cancer subtype was significantly (FDR < 0.05) associated with the TA heterocellular subtype, suggesting that luminal-B cancers might have a transitional phenotype between stem and differentiated cells, like TA in the colon-crypt. Interestingly, the basal-like and human epidermal growth factor receptor 2 (HER2)-enriched intrinsic breast cancer subtypes were significantly associated with the inflammatory heterocellular subtype (Fig. 1d and Supplementary Table 1e–g), suggesting increased immune phenotype in these subtypes. We further validated these results using the GSE42568 data set, with similar results (Supplementary Figure 1g and Supplementary Table 1h–j; the dominant subtype distribution in mixed/low-confidence samples is shown in Supplementary Figure 1h–j). This suggests that breast cancer subtypes are significantly (*p* < 0.05; Chi-squared test) associated with heterocellular signatures and explains additional characteristics of the intrinsic breast cancer subtypes.

### Luminal-A heterogeneity described by heterocellular subtypes

Surprisingly, the heterocellular signatures revealed the most heterogeneity in the relatively well-characterized luminal-A breast cancer subtype (Fig. 1d). This subtype was not only significantly associated with the differentiated goblet-like/enterocyte subtypes but, unexpectedly and interestingly, was also highly enriched for the poorly differentiated stem-like heterocellular subtype: 45% of luminal-A tumors were classified as stem-like tumors followed by 17% goblet-like, 15% enterocyte, 12% inflammatory, and 11% TA subtypes (Fig. 1e; *n* = 202). We further validated our results using an additional data set enriched for ER positive tumors (luminal-A; GSE6532^25–27^) observing similar high heterogeneity (Supplementary Figure 1k, l and Supplementary Table 1w–ab: tamoxifen-treated and -untreated samples; >39% stem-like, >24% inflammatory, >16% goblet-like, >8% TA, and >0.8% enterocyte subtype; the distribution of the dominant subtypes in mixed/low confidence and treated samples is shown in Supplementary Figure 1n). The proportions of inflammatory and enterocyte subtypes varied in the validation cohort, with the variable overall enterocyte subtype in luminal-A cancers from different data sets again representing that specialized colonic cells do not exist in breast cancers. Overall, we observed transcriptomic heterogeneity associated with heterocellular signatures in luminal-A breast cancer.

**Fig. 2:**
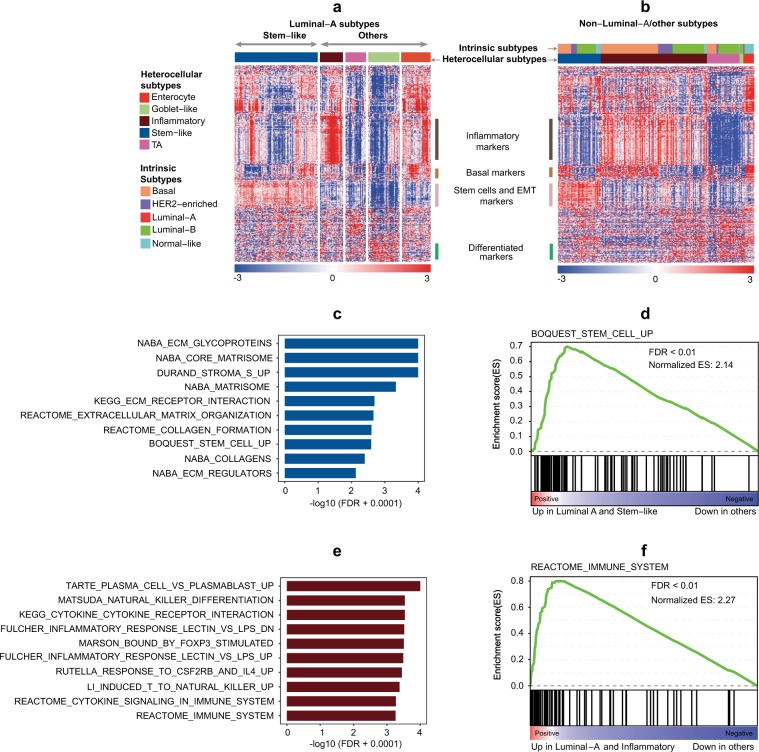
Heterocellular subtype-based heterogeneity in luminal-A breast cancers. **a**, **b** Heatmap showing the expression of the top highly variable and selected marker genes (371 genes from SD > 1.5 gene list shown in Supplementary Table 2e) between stem-like (*n* = 90) and other subtypes (*n* = 112) within the **a** luminal-A breast cancer subtype (*n* = 202; Supplementary Figure 1m) and **b** subtypes other than luminal-A (non-luminal-A) from TCGA breast cancer data^23^ (*n* = 205). **c**–**f** GSEA analysis showing gene sets enriched in **c**, **d** stem-like and **e**, **f** inflammatory heterocellular subtype samples compared with the other subtypes (*n* = 202; stem-like (*n* = 90), inflammatory (*n* = 25), other subtypes (*n* = 87)) from TCGA breast cancer.^23^ Relevant gene sets that were enriched were shown in **c**–**f** (See Supplementary Table 1k, l for the top gene sets that were ordered by significance of FDR values). KEGG—Kyoto Encyclopedia of Genes and Genomes; EMT—epithelial-to-mesenchymal transition

To further characterize these heterocellular subtypes in luminal-A breast cancers, we next performed heatmap analysis of heterocellular gene expression signatures using luminal-A and compared it to non-luminal-A (other subtypes) samples (Fig. 2a, b, Supplementary Figure 1m and Supplementary Table 2e). Here, our goal is to elucidate the heterogeneity in luminal-A using heterocellular subtypes. As expected, the goblet-like subtype contained increased expression of differentiated gene markers compared to the other heterocellular subtypes in luminal-A subtype (Fig. 2a). Although the TA subtype shared some of the differentiated gene markers, they showed increased heterogeneity similar to that of the CRC subtype,^11^ with 11% (*n* = 202; Fig. 1e) of the samples representing this subtype in luminal-A subtype.

Nevertheless, there was a consistent enrichment of the stem-like heterocellular subtype in luminal-A breast cancers, suggesting potentially interesting luminal-A characteristics. Of note, the stem-like subtype was enriched for potential luminal progenitor genes,^28^ with the presence of stem cell/epithelial-to-mesenchymal transition (EMT), myoepithelial, and basal cancer markers (Fig. 2a). We further confirmed this by geneset enrichment analysis (GSEA), which showed that the stem-like subtype of luminal-A cancers was enriched for stem and stromal fibroblast cells (Fig. 2c, d, and Supplementary Table 1k). Hence, luminal-A tumors represent heterogeneity at the heterocellular level.

### Immune heterogeneity in luminal-A tumors

**Fig. 3:**
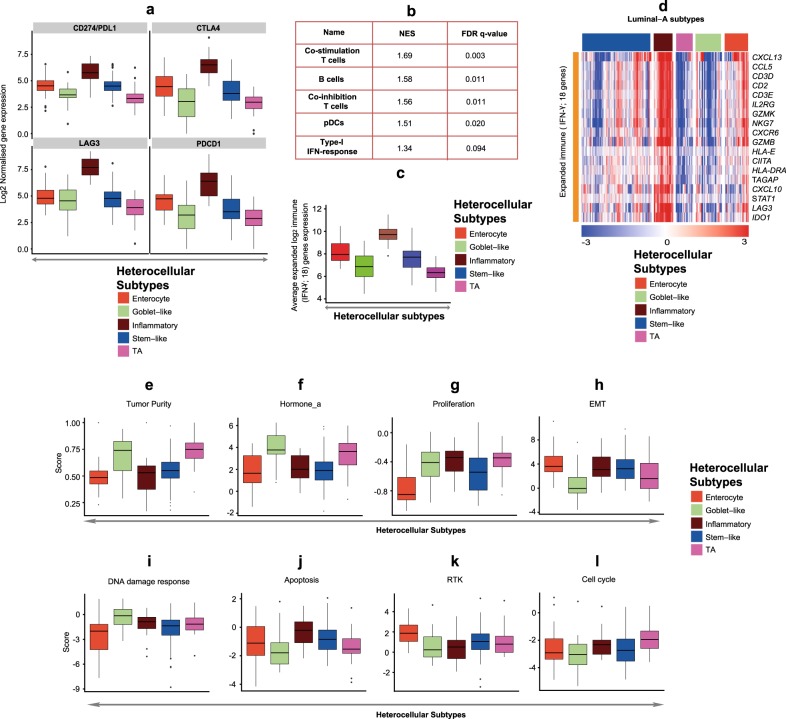
Enrichment of immune checkpoint genes, immune cells, expanded immune (18-gene) signature and other phenotypes in luminal-A heterocellular subtypes. **a** Box plots showing differences in the expression of immune checkpoint genes *CD274* (*PDL1), CTLA4*, *LAG3*, and *PDCD1* between heterocellular subtypes (*n* = 202; enterocyte (*n* = 31), goblet-like (*n* = 34), inflammatory (*n* = 25), stem-like (*n* = 90), TA (*n* = 22); TCGA^23^ breast cancer). Kruskal–Wallis test was performed to calculate *p* and their corresponding FDR values. Those associations with FDR < 0.05 was considered significant. **b** Gene set enrichment analysis (GSEA) showing immune cell types enriched in inflammatory heterocellular subtype samples compared to the other subtypes using the Rooney et al.^60^ gene sets (*n* = 202; inflammatory (*n* = 25) and other subtypes (*n* = 177); TCGA breast cancer^23^). Those associations with FDR < 0.1 was considered significant. **c** Boxplot showing differences in sample-wise average gene expression of 18 published expanded immune (18-gene) signature^29^ in heterocellular subtypes. Kruskal–Wallis test was performed to calculate *p* values. *p* < 0.05 was considered significant (*n* = 202; enterocyte (*n* = 31), goblet-like (*n* = 34), inflammatory (*n* = 25), stem-like (*n* = 90), TA (*n* = 22); TCGA^23^ breast cancer). **d** Heatmap showing the expression of eighteen published expanded immune (18-gene) signature^29^ genes between heterocellular subtypes from luminal-A breast cancers (*n* = 202; enterocyte (*n* = 31), goblet-like (*n* = 34), inflammatory (*n* = 25), stem-like (*n* = 90), TA (*n* = 22); TCGA breast cancer^23^). **e**–**l** Boxplots showing differences in **e** tumor purity, **f** hormone_a, **g** proliferation, **h** EMT, **i** DNA damage response, **j** apoptosis, **k** RTK, and **l** cell cycle scores from TCGA^23^ between heterocellular subtypes. The data from **f**–**l** were from RPPA data-based scores published by TCGA.^23^ Kruskal–Wallis test was performed to calculate *p* and their corresponding FDR values. Those associations with FDR < 0.05 was considered significant. pDCs—plasmocytoid dentric cells; NES—normalized enrichment score; FDR—false discovery rate; EMT—epithelial–mesenchymal transition; RTK—receptor tyrosine kinase

Although characterizing the immune gene expression heterogeneity in luminal-A tumors, we observed increased expression of immune pathways including chemokine signaling, cytokine–cytokine receptor interaction, immune system, and natural killer cell differentiation in inflammatory luminal-A subtype (Fig. 2e, f, and Supplementary Table 1l). Based on this pathway enrichment analysis, we hypothesized that the inflammatory subtype luminal-A cancers are enriched for the expression of immune checkpoint genes, potentially marking responses to immune checkpoint blockade. As expected, immune checkpoint genes and other immune markers were overrepresented in the inflammatory luminal-A cancers compared to the other subtypes (Fig. 3a). In addition, we observed increased enrichment of certain immune cell types in inflammatory luminal-A subtype (Fig. 3b). In order to predict if these inflammatory luminal-A tumors potentially may respond to anti-immune checkpoint therapy, we used a published ‘expanded immune gene’ signature, which potentially predicts anti-PD1 immune-checkpoint responses in melanoma and other cancers.^29^ All 18 expanded immune signature genes were highly expressed in the inflammatory subtype with increased average gene expression for the signature (Fig. 3c, d). Similarly, a proportion of the stem-like subtype showed increased expression of the immune genesets and expanded immune gene signature (Fig. 3d). These results suggest that luminal-A breast cancer subtype is heterogeneous with inflammatory heterocellular subtype showing exclusive immune infiltration.

### Additional characteristics of heterocellular subtypes

Next, we sought to understand if phenotypic changes that were measured as scores by TCGA^23^ show difference between our heterocellular subtypes in luminal-A tumors (Fig. 3e–l; scores in Fig. 3f–l are from reverse-phase protein microarray; RPPA as published by TCGA.^23^) Our analysis showed that tumor purity, hormone_a (represents signatures associated hormone receptors,^30^) proliferation and DNA damage response scores were significantly high in goblet-like and TA subtypes compared with the other subtypes (Fig. 3e–g and i). The inflammatory subtype showed high proliferation score similar to goblet-like and TA subtypes (Fig. 3g). On the other hand, the EMT and apoptosis scores were low in goblet-like and TA, but high in stem-like subtype (this subtype in CRC is known to have high EMT genes;^11^ Fig. 3h and j). We observed increased receptor tyrosine kinase score in enterocyte and stem-like subtypes and significantly increased cell cycle score in TA subtype (Fig. 3k and l). There were other phenotypes from the TCGA that were not significantly associated with the subtypes (Supplementary Table m). These results suggest that these heterocellular subtypes from luminal-A show differences in multiple breast cancer associated phenotypes.

### Association of heterocellular subtypes with other published luminal-A subtypes

**Fig. 4:**
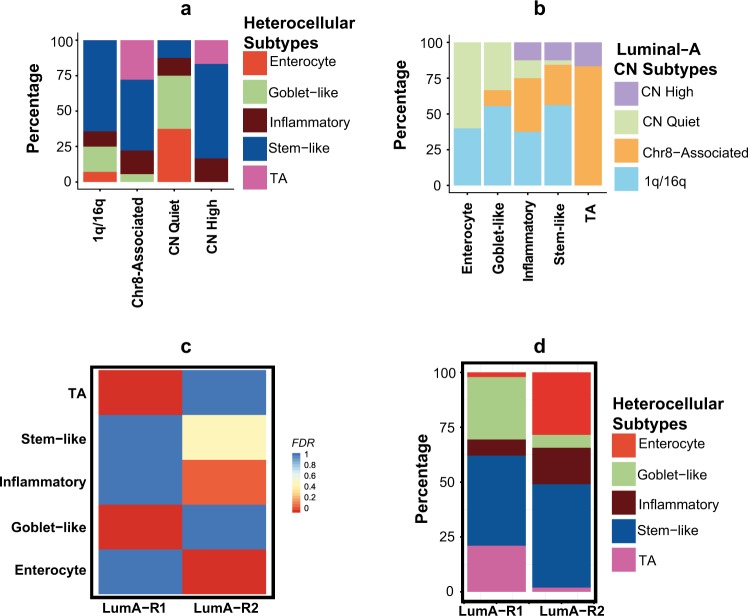
Association of heterocellular subtypes with published other luminal-A breast cancer subtype classifications. **a**, **b** Bar plots showing the percentage of **a** heterocellular subtypes in Ciriello subgroups of luminal-A subtype^3^ (1q/16q (*n* = 28), Chr8-associated (*n* = 18), CN quiet (*n* = 8), CN high (*n* = 6)) and **b** vice versa (enterocyte (*n* = 5), goblet-like (*n* = 9), inflammatory (*n* = 8), stem-like (*n* = 32), TA (*n* = 6), *p* < 0.02, Chi-square test; Supplementary Table 1n–p). We did not compare our heterocellular subtype to “mixed” subtype of Cirello, as it is reported to lack any decipherable patterns of chromosomal changes.^3^ Data for a-b) are from TCGA^23^ breast cancer. **c** Heatmap showing sample enrichment analysis using hypergeometric test-based FDR values comparing heterocellular subtype classification (*y* axis) with two Netanely et al.^32^ luminal-A breast cancer subtypes (*x* axis). **d** Bar plot showing percentage of different heterocellular subtypes in two Netanely et al., luminal-A breast cancer subtypes (LumA-R1 (*n* = 95), LumA-R2 (*n* = 102); *p* < 0.02, Chi-square test; Supplementary Table 1q–s). Only those samples classified into subtypes with high confidence from heterocellular subtype classification are shown in **a**–**d**

To understand potential mutational and CNA changes in heterocellular subtypes of luminal-A, we next compared our heterocellular luminal-A subtype classification with four Ciriello CNA-based luminal-A subtypes^3^ (Fig. 4a, b, and Supplementary Table 1n–p). Regarding the association of the heterocellular subtypes with Ciriello’s subtypes, the well-differentiated goblet-like and enterocyte subtype samples were primarily associated the Ciriello subtypes—1q/16q (characterized by 1q gain and 16q loss chromosomal regions) and CN quite (characterized by quite CNA spectrum). TA heterocellular subtype samples were primarily associated with Ciriello’s Chr8-associated (characterized by loss of 8p and gain of 8q chromosomal regions) subtype cancers, however, a certain proportion of them also represented CN high (CNH; characterized by multiple focal CNAs) Ciriello subtypes. The stem-like and inflammatory luminal-A subtype samples were heterogeneous and represented all the four Ciriello subtypes, and these subtypes had a scrambled genome such that 12.5% belonged to the Ciriello CNH subtype (Fig. 4b). Though there were associations between Ciriello and our heterocellular subtypes, these two represent different classification systems representing genetic and transcriptomic heterogeneity of the luminal-A subtype.

Similarly, we assessed Aure et al.^31^ and Netanely et al.^32^ luminal-A gene expression subtype classifications. Aure et al.^31^ subtype did not show any similarity to our heterocellular subtypes representing that these classification are quite different from each other (Supplementary Figure 2 and Supplementary Table 1t–v). This attributes to the fact that Aure subtypes were not exclusively based on luminal-A cancer samples. They show the enrichment of luminal-A cancer samples in two of their multi-level clusters.^31^ On the other hand, our heterocellular subtypes divided two of the Netanely et al.^32^ subtypes into sub-subtypes (Fig. 4c and d and Supplementary Table 1q–s). Netanely LumA-R1 was mainly divided into goblet-like and TA, whereas LumA-R2 was divided into inflammatory and enterocyte subtypes. Our stem-like subtype was not significantly associated with any of their two subtypes and substantially present in both the Natanely subtypes. This suggests that our heterocellular subtypes explain additional transcriptomic heterogeneity that these two previous subtype classifications did not reveal.

### Heterocellular luminal-A subtypes are associated with tamoxifen treatment-based clinical outcomes

**Fig. 5:**
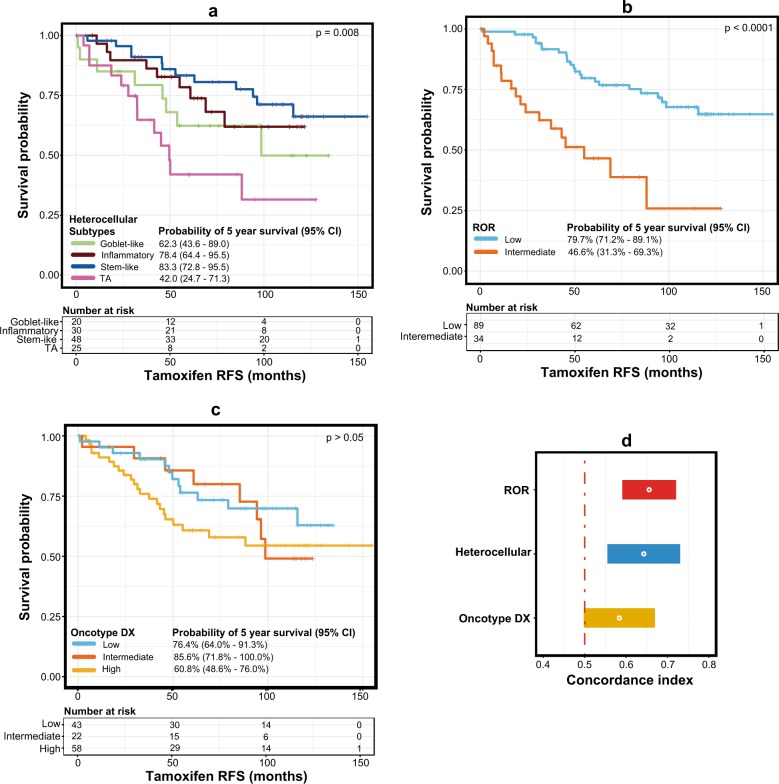
Survival differences in heterocellular subtypes from ER-positive tamoxifen-treated samples. **a**–**c** Kaplan–Meier survival curve showing tamoxifen-treated RFS (GSE6532;^25–27^ Supplementary Table 1w–y) between the **a** heterocellular subtypes, **b** groups from Risk of Recurrence (ROR,^34^) and **c** risk groups from OncotypeDX^35^ from ER-positive breast cancer samples from microarray data. **d** A plot showing concordance index and associated confidence intervals for RFS between heterocellular subtypes and ROR/OncotypeDX groups. Log-rank test was performed for the *p* values. RFS–recurrence free survival; CI–confidence interval

To assess the association of tamoxifen treatment response with heterocellular subtypes, we evaluated the association between our heterocellular luminal-A subtypes and clinical outcomes in patient samples treated with tamoxifen using GSE6532 data set^25–27^ (Fig. 5 and Supplementary Figure 3a; the distribution of the mixed/low confidence subtypes is shown in Supplementary Figure 1n). Heterocellular luminal-A subtypes showed significant (*p* < 0.01) differences in recurrence-free (RFS) and border-line significance (*p* = 0.07) differences in distant metastasis-free survival (DMFS) in patients treated with tamoxifen (Fig. 5a, Supplementary Figure 3b and Supplementary Tables 1w–y and 2f). We considered mixed subtype samples along with high confidence samples only in this case, and for mixed subtype samples only the dominant subtypes were considered. The consideration of mixed subtype was based on our previous report that mixed subtype tumors have a mixture of more than one subtype, and the presence of certain dominant subtype (for example TA) may attribute to prognostic and therapeutic response differences between subtypes/samples in CRC.^33^) Unlike in CRC,^11^ there was relatively good RFS and DMFS for luminal-A cancer patients with the stem-like subtype, similar to other subtypes including goblet-like and inflammatory subtypes. This may be attributed to the enrichment of expanded immune gene signature^29^ in a subset of stem-like subtype samples, similar to the immune-rich inflammatory luminal-A subtype with similar prognosis (Fig. 5a). Conversely, the TA subtype luminal-A tumors showed worse RFS and DMFS with tamoxifen treatment (Fig. 5a). Although there was a significant overall difference between subtypes for RFS/DMFS in tamoxifen-treated patients, there was no significant (*p* ≥ 0.5) difference in untreated patient samples (Supplementary Figures 3c, d, and Supplementary Tables 1z–ab and 2g). The lack of prognostic difference in the untreated patients but poor prognosis in treated patients with the TA subtype suggests that TA subtype luminal-A patients may respond less well to tamoxifen.

**Fig. 6:**
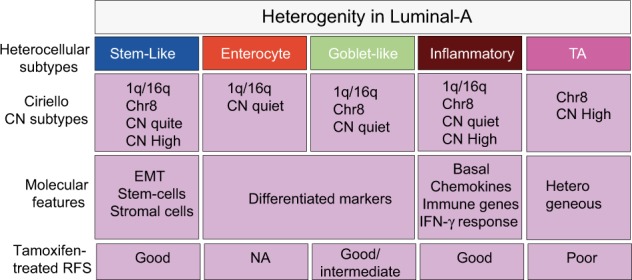
Summary of the luminal-A heterocellular subtypes and their characteristics. EMT—epithelial-to-mesenchymal transition; RFS—recurrence free survival; CN—copy number; chr-8—chromosome 8 associated; TA—transit amplifying; NA—not enough data available to conclude

We next compared these results with RFS from risk of recurrence (ROR^34^) and OncotypeDX.^35^ Among the three classifications, there was not much difference in RFS between ROR and our heterocellular subtypes, with similar concordance index (Fig. 5a, b, and d). However, the poor performance of OncotypeDX compared to our heterocellular subtypes could be attributed to the fact that the method was applied to microarray data (Fig. 5a, c, and d), which was not originally intended to be used. Nevertheless, these results warrant further validation using larger cohorts in the future. Overall, these results confirm the heterogeneity of luminal-A cancers and provide insights into the pathophysiology dictated by different cell types for potential personalized treatment (Fig. 6).

## Discussion

That breast cancers are heterogeneous is well known.^1,2^ Clinically, hormone receptor-positive breast cancer patients are treated differently to triple hormone receptor-negative (TNBC) and HER2-positive breast cancer patients.^36^ At the molecular level, breast cancer was one of the initial cancer types to be subtyped into intrinsic gene expression subtypes.^1^ Similar to clinical breast cancer subtypes, the molecular subtypes have distinct prognostic differences.^1,2^ In this study, we further investigated breast cancer heterogeneity, especially in the luminal-A subtype, using heterocellular subtype signatures defined in CRCs. This was done similar to the application of breast cancer subtype signatures to other cancers^21,22,37^ and with an intention to identify low frequency and unreported subtypes that are not apparent based on unsupervised approaches.

The basal subtype, which represents the majority of TNBCs, is already known to be highly heterogeneous, with the majority of these patients responding to chemotherapy.^38^ However, basal breast tumors often recur with aggressive disease.^39^ Similar to other studies,^40,41^ our results showed enrichment of immune genes characteristic in the basal/inflammatory breast cancer subtype (Fig. 1d). While no immunotherapy is yet approved, but with immune checkpoint inhibitors being tested clinically in patients with breast cancer,^42^ our association of a subset of basal breast cancers with the CRC inflammatory subtype suggests a means to identify patients who might respond to immunotherapy. This potentially aligns with responses to atezolizumab and pembrolizumab immunotherapy in metastatic TNBC patients.^43^ Our similar observation of an association between HER2 breast cancers and the inflammatory heterocellular subtype suggests that some HER2-positive patients may similarly be eligible for immunotherapy.

Moreover, we observed an enrichment of inflammatory heterocellular subtype samples in the luminal-A subtype harboring high expression of immune checkpoint genes. Next to the inflammatory subtype, there was a subset of the stem-like subtype with increased expression of immune genes (Fig. 3a and d). Both of these subtypes showed increased expression of expanded immune gene signature,^29^ suggesting potential response to immune checkpoint inhibition. Hence, our heterocellular gene signature may be useful for selecting patients within luminal-A breast cancers for immunotherapy, which warrants further exploration in the future. Although there are few indicators of how immunotherapy might work in relatively good prognostic luminal-A subtype cancers,^44^ tamoxifen-resistant TA luminal-A tumors do not seem to express many immune genes, suggesting that a combination of tamoxifen plus immunotherapy may not be the treatment of choice for resistant patients. Immune checkpoint inhibitors have now been approved for microsatellite instable CRCs, which are associated with the inflammatory CRC subtype.^45^ TA CRC tumors are enriched for microsatellite stable disease,^11^ suggesting potential resistance to immunotherapy. However, it may be interesting to find a way to induce this immune dormant TA luminal-A subtype to immune enriched subtype for potential immunotherapy.

Although the epithelial compartment of the breast and colon vary, we observed a significant association between luminal-A tumors and the goblet-like subtype, suggesting an overlap in common gene signatures representing a secretory function. Specifically, trefoil factor genes were highly expressed in both the luminal-A and goblet-like subtypes.^11,46^ Of note, the goblet-like luminal-A subtype enriched for the 1q/16q Ciriello subtype is associated with increased *KRAS* and *PIK3CA* mutations.^3^ We have previously shown that the CMS3 (goblet-like) subtype is enriched for *KRAS* mutations.^13^ In addition, a subset of TA and stem-like subtype luminal-A cancers was associated with the Ciriello CNH subtype, which is enriched for *TP53* mutations.^3^ Enriched *TP53* mutations also exist in TA and stem-like CRCs,^11,13^ suggesting that the subtype association between these cancer types is not random and they are associated with similar molecular events both at the transcriptomic and genetic levels. Again, this suggests that different cellular compartments share the same molecular features and perhaps functions. Nevertheless, the lower enrichment of the enterocyte heterocellular subtype in luminal-A cancers suggests the presence of this specialized cell type only in the intestine and not in the breast.

To our surprise, the stem-like subtype of luminal-A breast cancers showed good RFS (Fig. 5a), indicating that the presence of stem cells and fibroblasts (enriched in the stem-like subtype) does not indicate poor survival in differentiated luminal-A breast cancer patients, in contrast to CRC patients.^11,13^ On the other hand, the TA luminal-A subtype breast cancer patients showed poor RFS when treated with tamoxifen. However, none of these subtypes showed significantly different prognoses in the untreated patient samples. We recently developed a biomarker assay for CRC subtypes (both CRCAssigner and CMS) that stratify patients into subtypes^20,33^ and that potentially may select breast cancer patients for different therapies including immunotherapy. Overall, our current study sheds further light on luminal-A breast cancer heterogeneity that is useful for the personalized diagnosis and treatment of patients with luminal-A and other breast cancer subtypes.

## Methods

### Gene expression and patient survival data

The raw CEL files containing gene expression data and the corresponding survival data for patient tumors were downloaded from gene expression omnibus (GEO)^47^—GSE42568^24^ and GSE6532 (combined Affymetrix Human Genome U133A and U133B Arrays was used).^25–27^ Prognostic information for GSE6532 were from the original publications.^25–27^ The gene expression profiles for the TCGA breast cancer data (Ciriello et al.^23^) was downloaded from cBioPortal repository^48,49^ and other information of the corresponding samples were obtained from the original publication.^23^ Those genes with missing values (a value of zero from logarithmically transformed RSEM^50^ data) in >30% of the samples were removed, as described.^51^ Owing to the retrospective nature of this study using only publically available data, ethics approval for the study was not required.

### Affymetrix GeneChip® microarray data processing and quality control

The raw gene expression data (CEL files) were processed and normalized using robust multi-array normalization (RMA) from *R*-based Bioconductor^52^ package—*affy*.^53^ Only the samples having Normalized Unscaled Standard Error (NUSE;^54^ from *R*-based bioconductor^52^ *affyPLM*^55^ package) with a median score of 1 ± 0.05 was considered high-quality arrays and selected for further analysis GSE42568.^24^ For GSE6532^25–27^ (all samples were considered), data from two different arrays—Affymetrix GeneChip Human Genome U133A and U133B—done for the same set of samples were normalized using RMA^52^ and merged by samples. The technical/batch effect in GSE6532^25–27^ was corrected using ComBat.^56^ Supplementary Figure 3a shows a flow chart of the data processing and analysis for treated samples from GSE6532,^25–27^ which also applies for untreated samples.

### CMS and CRCAssigner classifications

For classifying the samples into CMS subtypes, *classifyCMS* function from our published R package *CMSClassifier*^13^ was used. We applied single sample prediction method from the package, and those samples that were classified as mixed or undetermined by *CMSClassifier* were considered mixed or low confidence samples, respectively (Supplementary Table 1a, b). For classifying the samples into heterocellular subtypes, the correlation of gene centroids for five subtypes and gene expression data using Pearson method from CRCAssigner subtypes and signatures was applied, as described previously.^11,13^ Before Pearson correlation analyses, we used our probe to gene mapping file from our original paper^12^ to map to the CRCAssigner PAM centroid genes. After correlation, those samples with maximum correlation coefficient among five of them <0.15 were considered low confidence samples and those with difference in correlation coefficients between first and second subtypes <0.06 were considered mixed samples as described previously.^13^ Only those samples qualified otherwise as high confidence samples were mainly considered for further analyses (Supplementary Table 1c, d). Only for GSE6532^25–27^ data analysis, high confidence, and mixed samples were considered. In this case of mixed samples, the dominant or the subtype with maximum correlation coefficient was considered for further analysis.

### Breast cancer intrinsic classification

The intrinsic breast cancer classification for GSE42568 data set was performed using an *R*-based Bioconductor^52^ package—*genefu*.^57^

### Reconciliation of subtypes

The association between the heterocellular and published intrinsic subtypes were performed using the hypergeometric test as described by us previously.^14^

### GSEA

This analysis was performed using standalone GSEA package from GenePattern^58^ using the c2 geneset from mSigDB^59^ and published immune cell specific gene markers from Rooney et al.^60^

### Visualization of gene expression data

For the heatmap, genes were clustered (hierarchical clustering) by Cluster 3.0^61^ using the default settings, followed by visualization of the clusters using *GENEE* from GenePattern.^58^

### Association between heterocellular subtypes and breast cancer phenotypes

Breast cancer phenotypes such as proliferation, apoptosis and other features as RPPA scores were from Ciriello et al.^23^ Association between these features and heterocellular subtypes were performed using Kruskal–Wallis statistical test and plotted as boxplots.

### Prediction of ROR/Oncotype DX risk groups

Prediction of tumor samples into ROR groups was performed as described.^34^ The OncotypeDX Recurrence Score were predicted as described.^35,62,63^ For microarray data, most variable probes were selected to represent the 21 OncotypeDX genes.^35^ *CD68* gene, which was not annotated in our data set, was replaced with its corresponding probe (*203507_at*). Five of the 21 OncotypeDX^35^ genes were housekeeping genes, whose average expression was subtracted from the other 16 OncotypeDX genes.^63^

### Survival analysis

Kaplan–Meier survival analysis was performed and concordance index was calculated using R package—*survival*^64^ and plotted using R package—*survminer*.^65^ For statistical test, log-rank test was used.

### Reporting summary

Further information on research design is available in the Nature Research Reporting Summary linked to this article.

## Supplementary information

Supplementary Information

Supplementary Table 1

Supplementary Table 2

Reporting Summary Checklist

## Data Availability

All the analyses in this study were done using R statistical software version 3.5.1. A list of packages used include *CMSClassifier*,^13^ for predicting CMS classes and CRCAssigner using our published classifier.^13,20^

## Supplementary information

**Supplementary Information** accompanies the paper on the *npj Breast Cancer* website (10.1038/s41523-019-0116-8).

## Data availability

The two Affymetrix gene expression profile data sets, GSE42568 and GSE6532, analyzed during the current study, can be accessed from the NCBI Gene Expression Omnibus (GEO) repository, https://identifiers.org/geo:GSE42568, https://identifiers.org/geo:GSE6532. Data set GSE42568 supports Fig. 1, Supplementary Figure 1 and Supplementary Tables 1 and 2 of the published article. Data set GSE6532 supports Fig. 5, Supplementary Figures 1 and 3, and Supplementary Tables 1 and 2 in this published article. The TCGA breast cancer data set analyzed during the current study can be accessed from the cBioPortal for Cancer Genomics repository, https://identifiers.org/cbioportal:brca_tcga_pub2015, and supports Figs 1, 2, 3 and 4, Supplementary Figures 1 and 2, and Supplementary Tables 1 and 2 in this published article. Additional information for the TCGA breast cancer data set was obtained from the published article 10.1038/nature11412. RNAseq data from normal breast cancer samples (cohort: GDC TCGA Breast Cancer (BRCA), gene expression data HTSeq−FPKM-UQ, *n* = 162 solid tissue normal) analyzed during this study, can be accessed from the University of California Santa Cruz (UCSC) Xena browser, https://xenabrowser.net/datapages/?cohort=GDC%20TCGA%20Breast%20Cancer%20(BRCA)&removeHub=https%3A%2F%2Fxena.treehouse.gi.ucsc.edu%3A443, and support Supplementary Figure 1 in this published article. The data analyzed during this study are described in the following data record: 10.6084/m9.figshare.8256713.^66^
