# Supplementary material for: Heterocellular gene signatures reveal luminal-A breast cancer heterogeneity and differential therapeutic responses
Source: NPJ Breast Cancer. 2019 Aug 2;5:21. doi: 10.1038/s41523-019-0116-8 (PMC6677833; doi:10.1038/s41523-019-0116-8)

Supplementary Information

Heterocellular gene signatures reveal luminal-A breast cancer heterogeneity and differential therapeutic responses

Pawan Poudel[1,*], Gift Nyamundanda[1,2,*], Yatish Patil[1,2,*], Maggie Chon U Cheang[3], and Anguraj Sadanandam[1,2,$]

[1] Division of Molecular Pathology, Institute of Cancer Research, London, United Kingdom

[2] Centre for Molecular Pathology, Royal Marsden Hospital, London, United Kingdom

[3] Division of Clinical Studies, Institute of Cancer Research, London, United Kingdom

* - Equal first authors

[$] Correspondence should be addressed to: Anguraj Sadanandam, Ph.D., Institute of Cancer Research (ICR), 15 Cotswold Road, Sutton, SM2 5NG, United Kingdom; anguraj.sadanandam@icr.ac.uk.

Supplementary Note

Association between heterocellular subtypes and adjacent normal breast tissue

We assessed the presence of the enterocyte and other cell type signatures in the normal adjacent breast tissue using our heterocellular subtypes and TCGA data[1-3] (n=25). Four subtypes were identified in the adjacent normal breast tissue, whereas enterocyte was underrepresented in this cohort. This suggests that the CRC specialized enterocyte signature is potentially not a representative of normal breast tissue. Nevertheless, we found significantly (FDR<0.05) increased percentage of normal breast tissue fat (defined by Troester et al.[3]) content in the stem-like and inflammatory subtypes compared to the other heterocellular subtypes from adjacent normal breast tissue samples, whereas the percentage of normal breast stromal content (defined by Troester et al.[3]) was low in both the subtypes (**Supplementary Figures 1a and b**). In contrast, the opposite was true in the goblet-like subtype. There was a high variability in TA subtype for both the fat and stromal percentages (**Supplementary Figures 1a and b**). On

the other hand, there was no significant difference in the percentage of normal epithelial content (defined by Troester et al.[3]) across heterocellular subtypes (**Supplementary Figures 1c)**. These results suggest differential presence of heterocellular subtypes in normal breast tissue and their association with normal fat and stromal contents. The lower variation in mature epithelial component compared to fat and stroma in the heterocellular subtypes of adjacent normal breast tissue suggest that our analysis was not biased towards the original derivation of this signature from CRC.

Supplementary Methods

Association of heterocellular subtypes and fat, stroma and epithelim in adjacent normal breast tissue.

RNAseq data ("HTSeq - FPKM-UQ") from adjacent normal breast samples[3] (n=162; represented as “Solid Tissue Normal” in phenotype.tsv file) were downloaded from University of California Santa Cruz (UCSC) Xena browser -- (https://xenabrowser.net/datapages/?cohort=GDC%20TCGA%20Breast%20Cancer%20(BRCA)&removeHub=https%3A%2F%2Fxena.treehouse.gi.ucsc.edu%3A443).

From the RNAseq data, those genes with missing values (a value of zero from logarithmic transformed RSEM data) in greater than 30% of the samples were removed, as described[4]. Heterocellular subtype classification was performed on this data. The features associated with fat, stroma and epithelium were a part of the Troester et al.[3] publication. The association between these features and heterocellular subtypes in adjacent normal breast tissue were calculated using Kruskal-Wallis statistical test and boxplots. Only 25 samples had all the features to perform the association analyses.

Supplementary Figure Legends

Supplementary Figure 1. Association of breast cancer and normal tissue with heterocellular subtypes. a-c. Boxplots showing percentage of a) fat, b) stroma and c) epithelium from adjacent normal breast tissue (from Troester, et al.[3]) present in different heterocellular subtype samples (n=25). **d**. Pie chart showing the distribution of the TCGA[1,2] (n=315) mixed samples by heterocellular subtypes. **e-f.** The proportion plots showing e) the maximum Pearson correlation coefficient and f) distances (the difference in correlation coefficients between the first and second subtypes) for each sample in the TCGA[1,2] dataset. Red horizontal dashed lines identify samples in e) with maximum correlation coefficient of less than 1.5 (the low confidence samples) or f) with distances of less than 0.06 (the mixed samples). **g.** Heatmap showing sample enrichment analysis using hypergeometric test FDR values comparing heterocellular subtypes (y-axis) with intrinsic breast cancer subtypes (x-axis) from GSE42568[5] (n=63; **Supplementary Tables 1H-J** and see Methods section). **h**. Pie chart showing the distribution of the GSE42568[5] (n= 41) mixed samples by

heterocellular subtypes. **i-j.** The proportion plots showing i) the maximum Pearson correlation coefficient and j) the distances (the difference in correlation coefficients between the first and second subtypes) for each sample in the GSE42568[5] dataset. Red horizontal dashed lines identify samples in i) with maximum correlation coefficient of less than 1.5 (the low confidence samples) or j) with distances of less than 0.06 (the mixed samples). **k-l.** Pie charts showing percentage of different heterocellular subtypes in estrogen receptor-positive (luminal-A) breast cancer samples k) treated [enterocyte (n=1), goblet-like (n=20), inflammatory (n=30), stem-like (n=48), TA (n=25)] and l) untreated [enterocyte (n=1), goblet-like (n=12), inflammatory (n=17), stem-like (n=24), TA (n=5)] with tamoxifen from GSE6532[6-8] dataset. Only those samples classified into high confidence and mixed subtypes are shown in **k-l**). The dominating (first) subtype was used for those samples showing mixed subtype identities. **n.** A pie chart showing how the mixed (not the low confidence) samples for the GSE6532[6-8] dataset (n=19) are distributed by heterocellular subtypes (**Supplementary Tables 1W-AB)**. **m.** Heatmap showing the expression of the top highly variable selected (standard deviation; SD>1.5) genes between different heterocellular subtypes within the luminal-A breast cancer subtype samples (n=202) from TCGA breast cancer[1,2] (**Supplementary Tables 2E**). A part of this figure is shown in **Figure 2a**.

Supplementary Figure 2. Comparison of heterocellular subtypes and Aure et al., clusters of luminal-A breast cancer. a. Heatmap showing sample enrichment analysis using hypergeometric test-based FDR values comparing heterocellular subtype classification (y-axis) with Aure et al., multilevel classification of breast cancer[9] showing luminal-A enriched clusters (x-axis). **b.** Bar plot showing percentage of different heterocellular subtypes in Aure et al., luminal-A enriched clusters[9]. The data for a-b) are from TCGA breast cancer[1,2] (n=91). Only those samples classified into subtypes with high confidence from heterocellular subtype classification are shown in a-b) (**Supplementary Tables 1T-V**).

Supplementary Figure 3. Flow chart of data analysis, proportions of and survival differences in heterocellular subtypes from estrogen receptor-positive samples. a. A flow chart showing a pipeline of data normalization and analysis for GSE6532[6-8] dataset for estrogen receptor-positive and tamoxifen-treated samples (n=176; **Supplementary Tables 1W-Y**). Similar approach was followed for tamoxifen-untreated samples (n=86). In the case of tamoxifen-untreated data, 86 samples were qualified as ER+ve samples, and among them 58 were qualified as high confidence + mixed heterocellular subtypes (see **Supplementary Tables 1Z-AB**). These were used for further analysis. **b.** Kaplan-Meier survival curve showing tamoxifen-treated samples with distant metastasis free survival (DMFS) between the heterocellular subtypes from estrogen receptor-positive breast cancer samples from GSE6532[6-8] dataset. **c-d.** Kaplan-Meier survival curve showing untreated samples with c) recurrence free survival (RFS) and d) DMFS between the heterocellular subtypes from estrogen receptor-positive breast cancer samples from GSE6532[6-8] dataset. Only those samples classified into high confidence and mixed

subtypes are shown in b-d). The dominating (first) subtype was used for those samples showing mixed subtype identities. RFS – recurrence free survival; DMFS – distant metastasis free survival; ER+ve – estrogen receptor positive; NR – not reached.

Supplementary Table Legends

Supplementary Table 1. A-D. Number and percentage of samples in CMS subtypes and heterocellular subtypes across two breast cancer datasets - TCGA[1,2] and GSE42568[5]. **E-J.** Number, percentage and FDR adjusted p values comparing the heterocellular subtypes with the intrinsic breast cancer subtypes in two breast cancer datasets - TCGA[1,2] and GSE42568[5]. **K-L.** Gene sets enriched in stem-like and inflammatory heterocellular subtypes of luminal-A tumors from TCGA[1,2] dataset. **M.** Nominal p and FDR values from different phenotypic characteristics of luminal-A breast cancer associated with heterocellular subtypes in TCGA[1,2] dataset. **N-V.** Number percentage and hypergeometric FDR values of samples associated with the comparison of heterocellular subtypes with the Ciriello *et al*[10]., Netanely[11] *et al*., and Aure *et al*[9]., luminal-A subtypes. **W-AB.** Number and percentages of ER-positive samples classified into heterocellular subtypes separately for treated and untreated samples from GSE6532[6-8]. FDR – false discovery rate.

Supplementary Table 2. A-B. CMS and **C-D.** heterocellular subtype identities or labels for TCGA[1,2] and GSE42568[5] datasets. **E.** Selected 371 genes (after SD>1.5) associated with luminal-A and heterocellular subtype from TCGA[1,2] dataset. **F.** Heterocellular subtype, ROR[12] and OncotypeDX[13] identities or labels for GSE6532[6-8] dataset for tamoxifen treated samples. **G.** Heterocellular subtype identities or labels for GSE6532[6-8] dataset for tamoxifen untreated samples.

Supplementary References

1 Cancer Genome Atlas, N. Comprehensive molecular portraits of human breast tumours. *Nature* **490**, 61-70, doi:10.1038/nature11412 (2012).

2 Ciriello, G., Gatza, M. L., Beck, A. H., Wilkerson, M. D., Rhie, S. K. *et al.* Comprehensive Molecular Portraits of Invasive Lobular Breast Cancer. *Cell* **163**, 506-519, doi:10.1016/j.cell.2015.09.033 (2015).

3 Troester, M. A., Hoadley, K. A., D'Arcy, M., Cherniack, A. D., Stewart, C. *et al.* DNA defects, epigenetics, and gene expression in cancer-adjacent breast: a study from The Cancer Genome Atlas. *NPJ Breast Cancer* **2**, 16007, doi:10.1038/npjbcancer.2016.7 (2016).

4 Hoadley, K. A., Yau, C., Wolf, D. M., Cherniack, A. D., Tamborero, D. *et al.* Multiplatform analysis of 12 cancer types reveals molecular classification within and across tissues of origin. *Cell* **158**, 929-944, doi:10.1016/j.cell.2014.06.049 (2014).

5 Clarke, C., Madden, S. F., Doolan, P., Aherne, S. T., Joyce, H. *et al.* Correlating transcriptional networks to breast cancer survival: a large-scale coexpression analysis. *Carcinogenesis* **34**, 2300-2308, doi:10.1093/carcin/bgt208 (2013).

6 Loi, S., Haibe-Kains, B., Desmedt, C., Lallemand, F., Tutt, A. M. *et al.* Definition of clinically distinct molecular subtypes in estrogen receptor-positive breast carcinomas through genomic grade. *J Clin Oncol* **25**, 1239-1246, doi:10.1200/JCO.2006.07.1522 (2007).

7 Loi, S., Haibe-Kains, B., Desmedt, C., Wirapati, P., Lallemand, F. *et al.* Predicting prognosis using molecular profiling in estrogen receptor-positive breast cancer treated with tamoxifen. *BMC genomics* **9**, 239, doi:10.1186/1471-2164-9-239 (2008).

8 Loi, S., Haibe-Kains, B., Majjaj, S., Lallemand, F., Durbecq, V. *et al.* PIK3CA mutations associated with gene signature of low mTORC1 signaling and better outcomes in estrogen receptor-positive breast cancer. *Proceedings of the National Academy of Sciences of the United States of America* **107**, 10208-10213, doi:10.1073/pnas.0907011107 (2010).

9 Aure, M. R., Vitelli, V., Jernstrom, S., Kumar, S., Krohn, M. *et al.* Integrative clustering reveals a novel split in the luminal A subtype of breast cancer with impact on outcome. *Breast Cancer Res* **19**, 44, doi:10.1186/s13058-017-0812-y (2017).

10 Ciriello, G., Sinha, R., Hoadley, K. A., Jacobsen, A. S., Reva, B. *et al.* The molecular diversity of Luminal A breast tumors. *Breast cancer research and treatment* **141**, 409-420, doi:10.1007/s10549-013-2699-3 (2013).

11 Netanely, D., Avraham, A., Ben-Baruch, A., Evron, E. & Shamir, R. Expression and methylation patterns partition luminal-A breast tumors into distinct prognostic subgroups. *Breast Cancer Res* **18**, 74, doi:10.1186/s13058-016-0724-2 (2016).

12 Parker, J. S., Mullins, M., Cheang, M. C., Leung, S., Voduc, D. *et al.* Supervised risk predictor of breast cancer based on intrinsic subtypes. *J Clin Oncol* **27**, 1160-1167, doi:10.1200/JCO.2008.18.1370 (2009).

13 Paik, S., Shak, S., Tang, G., Kim, C., Baker, J. *et al.* A multigene assay to predict recurrence of tamoxifen-treated, node-negative breast cancer. *N Engl J Med* **351**, 2817-2826, doi:10.1056/NEJMoa041588 (2004).

Supplementary Figure 1

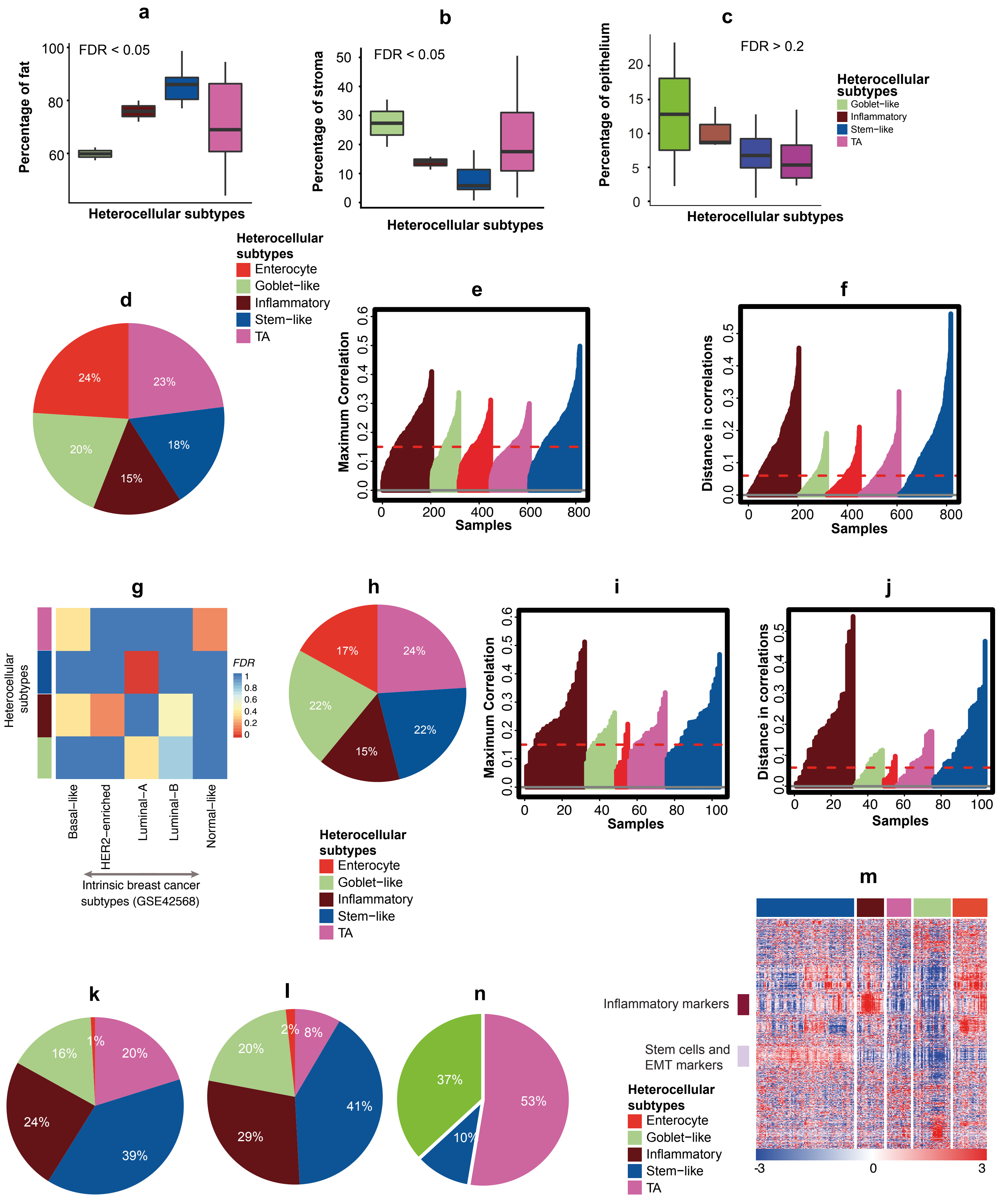

Supplementary Figure 2

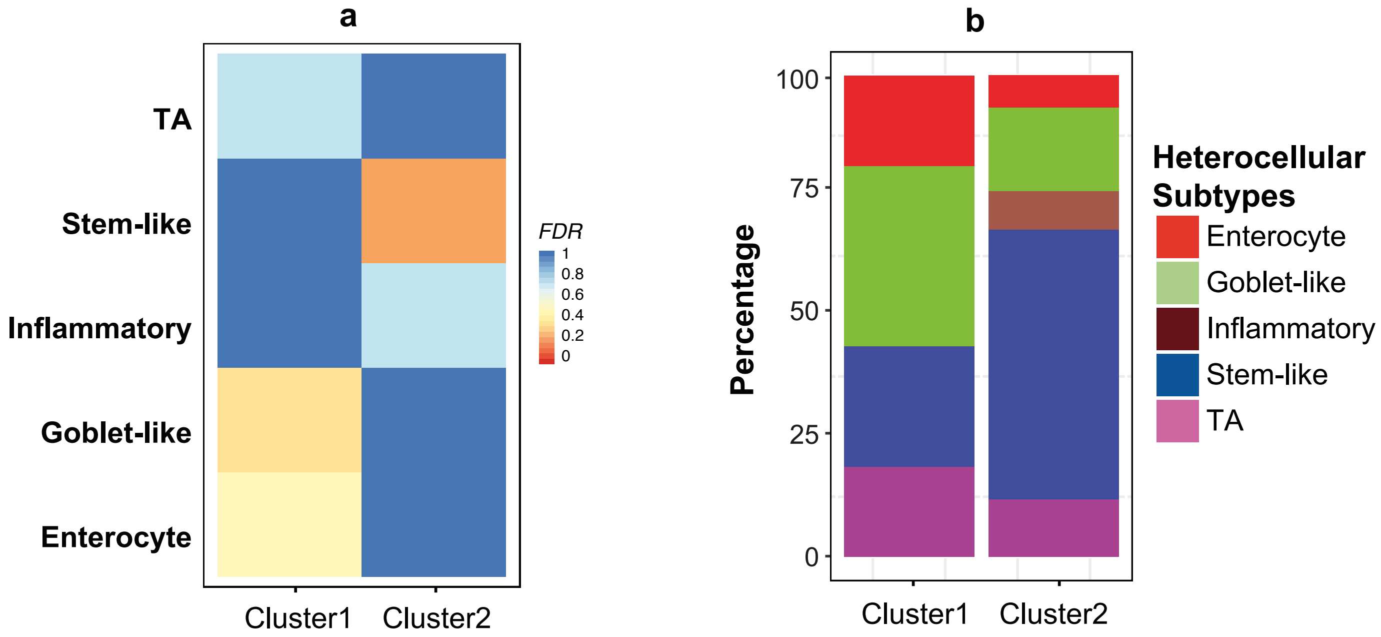

Supplementary Figure 3

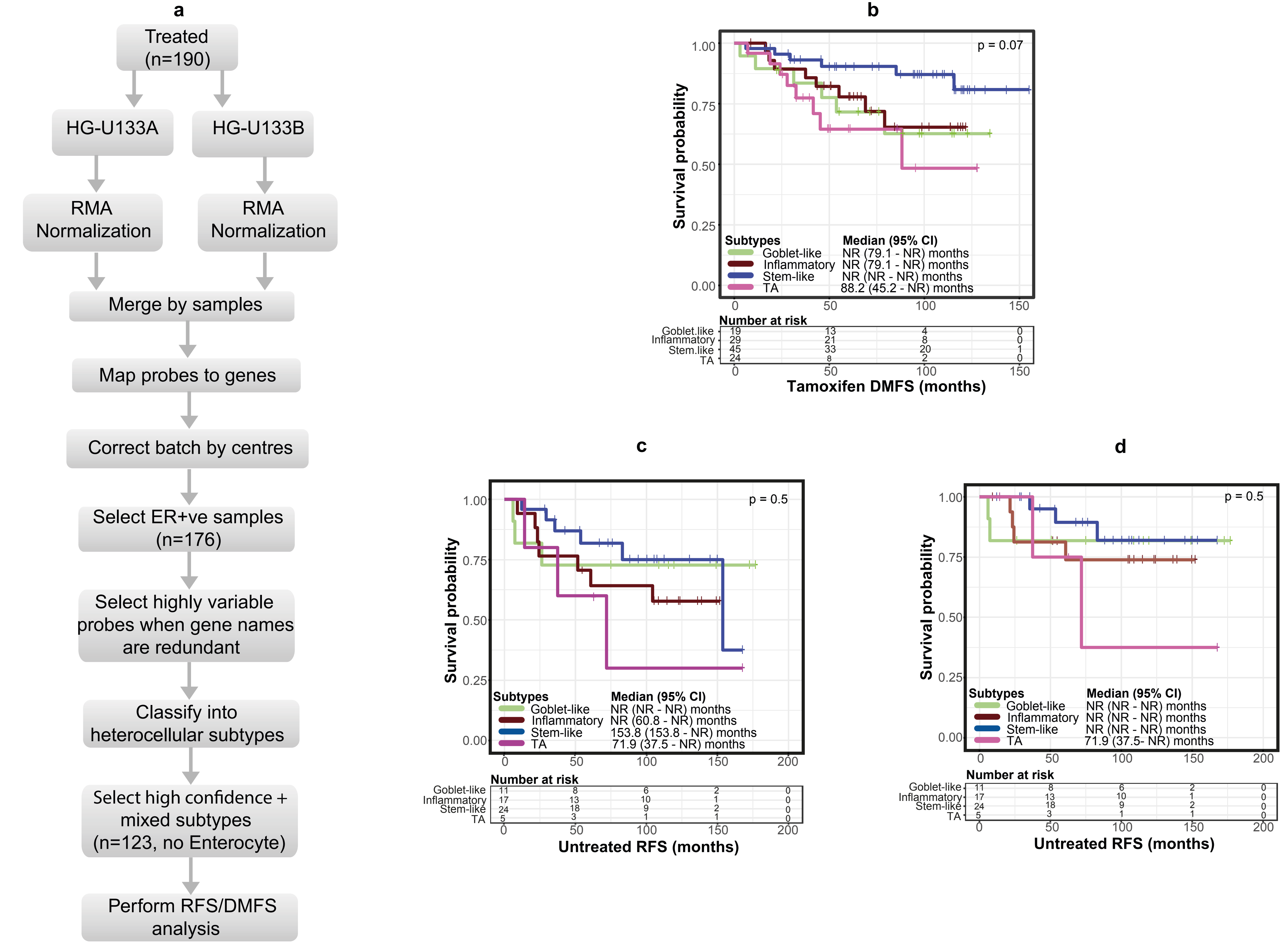

Supplement: Supplementary file 1 — Supplementary Information [file 41523_2019_116_MOESM1_ESM.pdf]
